# Measuring Domain-Specific Knowledge: From Bach to Fibonacci

**DOI:** 10.3390/jintelligence11030047

**Published:** 2023-02-28

**Authors:** Marianna Massimilla Rusche, Matthias Ziegler

**Affiliations:** Psychology Department, Faculty of Life Science, Humboldt Universität zu Berlin, 10117 Berlin, Germany

**Keywords:** domain-specific knowledge, knowledge domains, humanities, civics, science, CHC model, acquired knowledge

## Abstract

Along with crystallized intelligence (Gc), domain-specific knowledge (Gkn) is an important ability within the nomological net of acquired knowledge. Although Gkn has been shown to predict important life outcomes, only a few standardized tests measuring Gkn exist, especially for the adult population. Complicating things, Gkn tests from different cultural circles cannot simply be translated as they need to be culture specific. Hence, this study aimed to develop a Gkn test culturally sensitive to a German population and to provide initial evidence for the resulting scores’ psychometric quality. Existing Gkn tests often mirror a school curriculum. We aimed to operationalize Gkn not solely based upon a typical curriculum to investigate a research question regarding the curriculum dependence of the resulting Gkn structure. A set of newly developed items from a broad range of knowledge categories was presented online to 1450 participants divided into a high (fluid intelligence, Gf) Gf (*n* = 415) and an unselected Gf subsample (*n* = 1035). Results support the notion of a hierarchical model comparable to the one curriculum-based tests scores have, with one factor at the top and three narrower factors below (Humanities, Science, Civics) for which each can be divided into smaller knowledge facets. Besides this initial evidence regarding structural validity, the scale scores’ reliability estimates are reported, and criterion validity-related evidence based on a known-groups design is provided. Results indicate the psychometric quality of the scores and are discussed.

## 1. Introduction

In intelligence research, the integrated CHC theory of cognitive abilities (CHC model; e.g., Schneider and McGrew 2018) is a central and dynamic theory with regular updates of new or altered ability factors. Although by now a considerable number of other broad intelligence domains have been integrated into this CHC model, fluid intelligence (Gf) and crystallized intelligence (Gc) still are prominently featured due to the large number of studies supporting their utility with regard to predicting important life outcomes (Corley et al. 2012; Ghisletta et al. 2006; Kuncel et al. 2010). The CHC model combines Gc, reading and writing (Grw), quantitative knowledge (Gq), and domain-specific knowledge (Gkn) under the umbrella term acquired knowledge (e.g., Schneider and McGrew 2018). Gkn has been defined as “[…] *the depth, breadth, and mastery of specialized declarative and procedural knowledge (knowledge not all members of a society are expected to have)*”, (Schneider and McGrew 2018, p. 117). Recent theories such as the PPIK theory of adult intellectual development (intelligence-as-Process, Personality, Interests, and intelligence-as-Knowledge Model; Ackerman 1996, 2000) or OFCI (Openness-Fluid-Crystallized-Intelligence Model; Ziegler et al. 2012, 2015; Trapp et al. 2019) emphasize the importance of domain-specific knowledge by suggesting that the complex interplay between interests, personality, and Gc manifests in differences in Gkn. Moreover, Gkn has been shown to predict important life outcomes (e.g., Alexander and Judy 1988; Ackerman and Rolfhus 1999). Unfortunately, only few standardized tests measuring Gkn exist. Moreover, many of the existing tests mirror the content of school curricula (e.g., Schipolowski et al. 2020; Wilhelm et al. 2014). Consequently, the current study was conducted to derive a Gkn test which is culturally sensitive to a German population and to provide initial evidence for the resulting scores’ psychometric quality. To further investigate how far structural validity of scores from such tests is sensitive to school curricula, we aimed to operationalize Gkn not solely based upon a typical curriculum and to explore whether the resulting Gkn structure would differ from these approaches. Thereby, the study contributes to the breadth and scope of cognitive ability tests by showcasing the development of a test focusing an often neglected but important ability.

### 1.1. Theoretical Models of Domain-Specific Knowledge

Rolfhus and Ackerman (1996, 1999); (Rolfhus 1998) developed a Gkn test for the USA based on items from the US College Board. Their test comprises 20 knowledge categories which are mostly academic. An exploratory factor analysis (EFA) showed that these categories can be structured into four knowledge domains with a higher order Gkn-factor. Building upon this Gkn structure and test von Stumm (2013) developed a Gkn test for the UK population. In contrast to the US test, it also includes categories representing modern everyday life, such as Film or Sports. An EFA revealed a structure of two broad factors. Von Stumm classified these as Popular Knowledge and Academic Knowledge. Overall, these findings suggest a hierarchical Gkn structure with knowledge categories loading on a set of correlated latent variables (domains), which load on a higher order factor. This hierarchical Gkn structure can be assumed to be generalized, as it can be found across cultures. Following Steger et al. (2019), the factor structure of declarative knowledge and its interpretation highly depends on the compilation of the knowledge categories and items. In a smartphone-based assessment of 34 knowledge categories, the broadness of the sampling showed to be particularly relevant as the results showed that such broader knowledge category samples will also reveal a higher number of knowledge factors. 

Based on this, we aimed to create a broad Gkn test focused on German culture-specific content in order to add a non-proprietary alternative to other Gkn measures developed for the German population. A prominent example is the BEFKI (Berliner Test zur Erfassung Fluider und Kristalliner Intelligenz (Berlin Test for the Assessment of Fluid and Crystallized Intelligence); Schipolowski et al. 2020; Wilhelm et al. 2014). The BEFKI is a well-established German intelligence test designed for children and adolescents including one scale that assesses declarative knowledge as a Gc indicator. This knowledge scale comprises the knowledge domains Sciences, Humanities, and Social Studies. The BEFKI operationalizes Gkn based on items mostly following a typical school curriculum. However, beyond the BEFKI, there is a scarcity of comprehensive German culture-specific tests looking at Gkn. Other Gkn tests cannot simply be translated as they need to be culture specific. Hence, since it is favorable to have different instruments available to capture a construct, our aim was to develop an additional comprehensive German Gkn test based on a different item development approach than the BEFKI. In addition, the new test was conceptualized more broadly by including knowledge categories and respective items not solely based upon a school curriculum. This way, the current results can shed light onto the curriculum sensitivity of the Gkn structure. 

Besides structural theories, there is work regarding a relation between acquired knowledge such as Gc/gc and Gf/gf. Cattell’s (1943, 1987) investment theory assumes a dynamic relation between both intelligence factors, with gf having a permanent influence on gc across the life span (e.g., Ziegler et al. 2015; Beier and Ackerman 2001). These robust findings will be used in this study to inform the design of a known-groups validation (e.g., Cronbach and Meehl 1955; Hattie and Cooksey 1984) aimed at providing criterion-related validity evidence. Specifically, based on the investment hypothesis as well as assumptions about the relationship between Gf and Gkn as reflected in the CHC, OFCI, and PPIK models (e.g., Ackerman 1996; Beier and Ackerman 2003; Schneider and McGrew 2018; Ziegler et al. 2012), we assume that participants with a high level of Gf will overall show significantly better Gkn test results than participants with an average level of Gf. We expect the greatest difference in the Science domain due to the complex and abstract nature of science knowledge (e.g., Gabel 1999; Millar 1991).

### 1.2. Construction of a New Gkn Test

**Figure 1:**
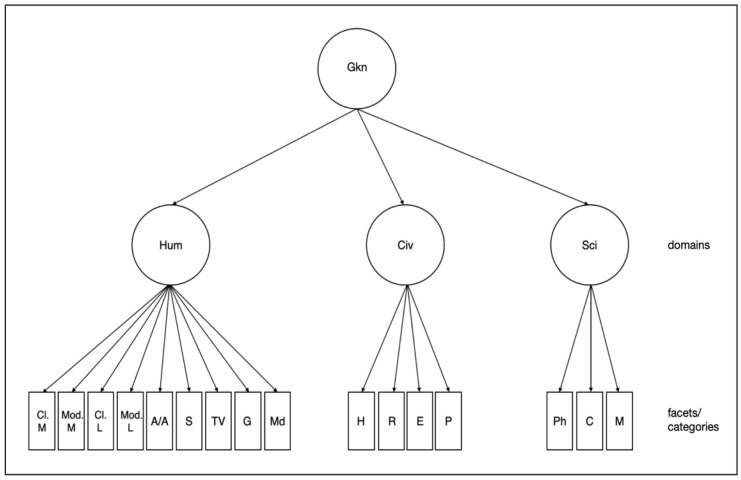
Hierarchical Structure of the Domain-specific Knowledge Model. Gkn = Domain-specific Knowledge, Hum = Humanities, Civ = Civics, Sci = Science, Cl. M = Classical Music, Mod. M = Modern Music, Cl. L = Classical Literature, Mod. L = Modern Literature, A/A = Art/Architecture, S = Sports, TV = Television, G = Geography, Md = Medicine, H = History, R = Religion, E = Economics, P = Politics, Ph = Physics, C = Chemistry, M = Mathematics.

The broad strokes of the theoretical model underlying the new Gkn test named the DoKnow Test *(Domain-specific Knowledge Test*) are mainly based on the Ackerman and Rolfhus’ Gkn model. Consequently, we also assumed a hierarchical structure with a Gkn-factor at the top and three narrower factors below which each can be divided into smaller facets. We aimed at including a large number of those facets as this was shown to be of importance (Steger et al. 2019). At the same time, such a larger number would increase the risk of not finding only three second-order factors. Thus, this approach will test the curriculum sensitivity of the Gkn structure. This idea of a hierarchical structure also corresponds to Schroeders et al. (2021) who proposed a hierarchical model of declarative knowledge comprising a gc factor as declarative knowledge at the top and broad knowledge areas below followed by various smaller knowledge domains. At the lowest hierarchy level, the model comprises nodes/nuances representing variations on a specific topic. The detailed Gkn model which resulted here is displayed in Figure 1.

On facet level, the DoKnow Test was created by integrating Rolfhus and Ackerman’s facets/knowledge categories, based on a school curriculum—such as Geography and Chemistry—with facets of von Stumm’s “Popular Knowledge” factor focusing on everyday life—such as Sports and Film. Moreover, all constructed items were not only fit into those facets but also chosen to fit into a German cultural background. By combining both the school curriculum and the everyday life approach, a much broader content was created. Moreover, the items are not mere translations but contain culture-specific knowledge. Thus, despite the parallels on facet level, it was an open question whether the facet scores could be aggregated into knowledge domains similar to Ackerman and Rolfhus’ model which comprises the four domains of Humanities, Civics, Science, and Mechanical. It is also important to acknowledge that, taking the test duration into account, we decided on a Technology category instead of a whole Mechanical domain in order to add more everyday life categories whose content was mainly derived from popular quiz outlets such as TV shows and quizbooks. However, the Technology category was excluded from the Gkn test in the further course of the test development due to poor psychometric quality based on CFA model fit indices and factor loadings. With regard to the specific item content, all items were newly developed and rated by four psychological experts to ensure them being culture specific and likely to be age invariant. In sum, item construction was based on existing theoretical models. However, the combination of curriculum-based knowledge and popular knowledge along with the cultural adaptation raised the question whether the hierarchical structure found in other tests, which either reflect only curriculum knowledge or a different cultural background, could be found.

A structural comparison of the three tests is presented in Table 1. Overall, this new Gkn test is intended to be mainly used for open science research targeting the general German public. 

### 1.3. Validation Strategy

The first evidence for the test scores’ construct validity has been established (Rusche and Ziegler 2022). Based on the theoretical assumptions detailed above, the following hypotheses were delineated to provide evidence for the test scores’ reliability and validity regarding structure and criterion-related validity.

1. The data collected, reflecting Gkn, are arranged in a hierarchical structure with one factor at the top and three narrower factors below, which each can be divided into smaller knowledge facets.
2. Criterion-related validity evidence will be provided based on a known-groups design. Participants with higher Gf scores will show overall significantly better test results than the unselected Gf participants (known-groups comparison), based on Cattell’s investment hypothesis. The greatest difference is expected in the Science domain due to the complex and abstract nature of Science knowledge. The reliability of the test scores will be estimated.

## 2. Method

### 2.1. Participants

The total sample comprised 1450 participants divided into a subsample of high Gf individuals and a subsample of individuals from the general population (unselected Gf sample). In this context, high Gf is defined as IQ 130+ measured in a Gf test. Following the idea of using Mensa membership as a high Gf-proxy (e.g., Bessou et al. 2004; Dijkstra et al. 2011; Egeland 2019; Fogel 1968; Storek and Furnham 2012), participants of the first group were members of Mensa Germany e.V., the largest German society for individuals with IQ 130+. The 415 participants (51% female) of this group have a mean age of 39.7 (*SD* = 11.4); the 1035 participants (61% female) of the second group have a mean age of 39.4 (*SD* = 15.3). Overall, age ranged between 15 and 92 years. Scores form the DoKnow Test were also investigated with regard to their relations to age, interests, and investment traits (Rusche and Ziegler 2022). Although the participants of the second group did not complete a Gf test, several studies have shown that cognitive ability scores exceeding an IQ of 130 are very rare even in selected groups. For example, Ziegler et al. (2009) tested 271 psychology students with a median age of 20 who achieved a mean IQ score of 117. Similarly, Iqbal et al. (2021) tested 100 medical students aged 20–21 years of which only 3 students achieved an IQ score higher than 120.

As their highest educational attainment, 46.84% of the unselected Gf participants stated to have an academic degree, 23.77% stated a general university entrance qualification, whereas 9.76% stated an advanced technical college entrance qualification. Moreover, 17.69% stated an intermediate school or lower secondary school leaving qualification, whereas 1.93% stated no educational degree. From the high Gf sample, 68.43% of the participants stated to have an academic degree, 20.24% stated a general university entrance qualification as highest educational attainment, whereas 3.86% stated an advanced technical college entrance qualification. In addition, 6.27% stated an intermediate school or lower secondary school leaving qualification, whereas 1.20% stated no educational degree. 

All participants were required to have German nationality and have lived in Germany from an early age.

### 2.2. Procedure

The study was designed as an online power test. Participants were recruited via social media and mailing lists to include numerous professions and avocations. To reach high Gf persons, Mensa Germany e.V. was contacted directly. The study took an average of 30 min. Participants had the possibility to request individual feedback. The present study adhered to the American Psychological Association’s (APA) Ethical Principles of Psychologists and Code of Conduct. Due to the absence of customary ethics board approval in the institute where the research was conducted, the aforementioned ethical guidelines were followed to ensure the protection of participants’ rights and well-being.

### 2.3. Measures

The test battery included the newly constructed items for the DoKnow Test and additional demographic items. To create the DoKnow Test, various items were constructed, fitting to the knowledge categories and domains previously chosen. Four psychological experts rated the items with regard to cultural fit, difficulty, invariance across age groups, and category fit to make sure that overlap among categories was avoided. They also discussed disagreements on the correct item answers and came up with a generally agreed upon solution for each item. To test numerous items, a planned missingness design (Little and Rubin 2019) was used. Hence, five different test versions were specified, and each participant received one. Each version contained the same 19 knowledge categories with a mix of unique and shared items. Two unique items per category were created for each of the five test versions. Additionally, all test versions contained one identical shared item (“linking item”) for each category in order to create a link between the test versions. Hence, all participants received one identical item per category. These linking items allowed to estimate missing information based on multiple imputation. Based on this, it was possible to perform further statistical calculations. All items were open-ended questions asking for a name, number, or short term (see Table 2 for examples). Altogether, there were 209 items: 5 test versions * 19 categories * 2 unique items per category + 19 shared items across all versions. 

### 2.4. Statistical Analyses

Statistical analyses were conducted using R (R Core Team 2016–2019) in R Studio (RStudio Team 2016–2019), especially the mice package (van Buuren and Groothuis-Oudshoorn 2011) and the lavaan package (Rosseel 2012). 

First of all, the answers for each item were gathered. Wrong answers were identified for each item by hand due to the open-ended response format. Thus, a criterion regarding spelling mistakes was set. It comprised the question whether an answer would be identified as correct in an oral exam situation. In this manner, each answer was classified into correct or incorrect which was also discussed in an expert group in case of inconclusive answers. This process was also used for data cleansing; data from 69 participants were removed due to non-German nationality, missing descriptive information, or nonsensical answers. Afterwards, all answers were numerally coded with either 1 (=correct) or 0 (=incorrect). As each participant received just one of five test versions, there were 80% of planned missing data with regard to the 190 unique items. However, due to the shared items it was possible to create a link between all test versions in this missing completely at random multi-matrix design and then use multiple imputation to estimate missing values with a “multivariate imputation by chained equations” (MICE; Gibbs sampling) technique. First, several replacement values for the missing data were imputed (5 estimates per variable). In this context, each incomplete variable is imputed by a separate model, modelled as a function of the other variables in the data. Subsequently, estimates from each model are pooled into a single set of estimates completing the data set. It has been shown that missing completely at random ensures that imputations are accurate and efficient (Lawes et al. 2020; Scheffer 2002) and is particularly valuable in factor analytical approaches (Revelle et al. 2017). 

After the imputation, the data were ready for numerous confirmatory factor analyses (CFA) used because a clear theoretical model was assumed to underlie the data. It is important to stress here that this hierarchical model reflects research, mostly based on test data derived from items mirroring school curricula. Thus, model fit here would indicate that the structure of Gkn is not sensitive to this. All in all, 57 CFAs were conducted to examine each category model (M_cat1_–M_cat19_) for both subsamples and the total sample with the newly developed items as indicators to confirm that each subscale is unidimensional (Ziegler and Hagemann 2015). For these CFAs, the robust weighted least squares estimator (WLSMV) was used. In addition, McDonald’s ω was computed as reliability estimate. The model fits of the knowledge category subscales were also used as criterion to decide whether items needed to be excluded from the respective category subscale. Model fits of the category models M_cat1_–M_cat19_ were evaluated against the Chi-Square Goodness-of-Fit statistic and other fit indices, comprising the Comparative Fit Index (CFI; Bentler 1990), Root Mean Square Error of Approximation (RMSEA; Browne and Cudeck 1993; Steiger and Lind 1980), and Standardized Root Mean Squared Residual (SRMR; Bentler 1995). Acceptable model fits were indicated by the following cut-offs (acceptable fits were seen as sufficient due to the subscales’ heterogeneous content): CFI > .90, RMSEA < .06, and SRMR < .08 (Hooper et al. 2008; Hu and Bentler 1999). Single items and subscales with poor psychometric quality—whose inclusion kept fit indices above cut-offs—were excluded subsequently.

In addition to these CFAs on category level, further CFAs were conducted for the three content domain models (M_dom1_–M_dom3_) to confirm the assumed structure using the category factor scores as indicators. In this case, the robust maximum likelihood estimator (MLR) was used. Again, McDonald’s ω was computed. Additionally, correlations between latent domain factors were calculated. Finally, different structural models (M_Gkn1_, M_Gkn2_) comprising all knowledge domains were tested with the domain factor scores as indicators. Subsequently, a statistical comparison between the different model types was conducted to examine whether the collected data can indeed be described better with the hierarchical model (M_Gkn1_) than with a one-factor model (M_Gkn2_). Both models were tested with CFAs using a MLR estimator and compared using a Chi-Square Difference Test (Satorra and Bentler 2001) and comparing AIC values (Akaike Information Criterion; Akaike 1974). Additionally, McDonald’s ω was calculated. 

To examine criterion-related validity of the DoKnow Test, latent mean differences between the high Gf and the unselected Gf sample were tested as a known-groups comparison. However, to find out first whether the DoKnow Test can be applied equally, it was necessary to determine if the test was measurement invariant across both samples. For this, multi-group CFAs for the hierarchical Gkn model were performed across samples to sequentially test configural invariance, metric invariance, and scalar invariance. Following Chen’s (2007) recommendations on cut off points for measurement invariance model fit indices with a sample size of n > 300, cut off points for metric invariance were set at a change of ≥−.010 in CFI supplemented by ≥.015 in RMSEA or ≥.030 in SRMR. To assume scalar invariance, cut off points were set at a change of ≥−.010 in CFI, supplemented by ≥.015 in RMSEA or ≥.010 in SRMR. After measurement invariance at a scalar level was confirmed, latent mean differences for each domain and the Gkn-factor between both samples were examined and the effect size Cohen’s *d* (Hancock 2001) was calculated at this latent variable level.

## 3. Results

### 3.1. Structural Validity

#### 3.1.1. Descriptive Statistics and CFAs of Knowledge Categories

Descriptive statistics of the knowledge categories, their final categorization to a knowledge domain, and omegas are given in Table 3. Initially, 19 categories and 209 items were included in the test. CFAs showed good model fits for 16 categories indicating unidimensionality. However, three categories (Popular, Biology, Technology) and 72 items (33 items belonging to those three categories plus 2–3 items per remaining category) were excluded due to poor psychometric quality. Hence, the final test consists of 16 categories and 137 items. Category scores are computed by summing up item scores (1 or 0) and calculating an average. 

Model fit indices of each final knowledge category are presented in Table 4 (total sample), and Tables S1 (high Gf) and S2 (unselected Gf) in the Supplementary Materials (SM). With a median of .246 and a mean of .307, item loadings in the total sample ranged from .028 to .989. Both the lowest and highest loading items are part of the Politics category. In addition, correlations between the categories for all samples are presented in Tables S3 and S4 in the SM. With a median of *r* = .19, the lowest correlation in the total sample was between the Chemistry and Modern Literature categories (*r =* .01), whereas the highest correlation was between Chemistry and Physics (*r =* .52). 

#### 3.1.2. CFAs of Knowledge Domains

CFAs proved a good fit supporting the presumed structure of three domains as all 16 categories could be assigned to a specific domain. Only two categories showed to be subordinated to a different domain than hypothesized (Medicine = Humanities instead of Science, Religion = Civics instead of Humanities) based on comparing the domains’ fit indices including and excluding the respective categories. Omegas ranged between .61 and .73. Comparing model fits of the total sample, the Civics (CFI = 1) and Science (CFI = .998) domains showed very good fits. The model fit of the Humanities domain (CFI = .943) was somewhat worse. Fit indices and omegas of each knowledge domain for all samples are presented in Table 5. In addition, correlations between the latent domain factors are presented in Table 6.

#### 3.1.3. CFAs of Gkn Models

Model fits of the two alternative Gkn models for all samples are presented in Table 7. The hierarchical model showed a significantly better fit than the one-factor model (Δχ^2^(*N* = 1450, 5) = 237.12, *p* < .001; total sample). Likewise, the hierarchical model showed the lowest AIC value.

#### 3.1.4. Factor Loadings on Knowledge Domains and the Gkn-Factor 

Loadings for all knowledge categories on their respective domain and loadings for all domains on the Gkn-factor are shown in Table 8. All loadings were significant (*p* < .001). In the total sample, loadings for Humanities ranged from .299 to .646, loadings for Civics from .464 to .580, and loadings for Science ranged from .553 to .614. The loading for Civics on the Gkn-factor had to be fixed to 1 and the residual variance of Civics to 0 in order to avoid a negative residual variance, while Humanities loaded highly on the Gkn-factor with .858. In contrast, Science loaded lower with .545. 

#### 3.1.5. Measurement Invariance Analyses between High Gf and Unselected Gf Samples

Model fit statistics for the configural, metric, and scalar levels of measurement invariance between both samples are presented in Table 9. Although the SRMR index increases with the constraints, the CFI decreases and the RMSEA does not change, so that scalar invariance was assumed following Chen’s (2007) recommendations detailed above. This result allows a latent mean comparison between both samples. 

### 3.2. Criterion-Related Validity

#### Mean Differences of Test Performance 

Table 10 presents latent means and mean differences between both samples and their effect sizes Cohen’s *d* showing significant differences in the Civics and Science domains and the Gkn-factor favoring the high Gf sample. The largest difference was found in Science. 

## 4. Discussion

This special issue aims at portraying the breadth and scope of cognitive assessment in the 2020s. As it stands, many cognitive ability tests focus on fluid or reasoning abilities on the one hand and crystallized ability (e.g., vocabulary) on the other hand. However, theories describing the structure of intelligence often contain the notion of acquired knowledge. Here, we have focused this concept as defined in the CHC model. The aim of the current research project was to develop an open-source Gkn test sensitive to German culture. Tests like this one are relevant to cover the ability spectrum more comprehensively. To this end, curriculum-based knowledge and popular knowledge was operationalized following the theoretical models suggested by Rolfhus and Ackerman, and von Stumm. This also allowed to test whether the assumed hierarchical Gkn structure can also be found in a German culture, and with a broader content than just curriculum-based knowledge. The current findings corroborate a hierarchical factor structure with a single Gkn-factor at the top and three lower order knowledge domain factors which again can be divided into several facets. In addition to the structural validity evidence, the current study also provides criterion validity related evidence by using a known-groups design. Here, it could be shown that a theoretically assumed mean difference between gifted and non-gifted persons exists. In combination with the reliability estimates and prior validity evidence (Rusche and Ziegler 2022), there is sufficient evidence to support the use of the DoKnow Test in research settings.

### 4.1. Hierarchical Structure of Gkn

We wanted to test the structural sensitivity of Gkn models by including a large portion of items not based on a school curriculum. The findings support the notion of a hierarchical Gkn model rather than a one-factor model. In particular, the hierarchical structure with three domain factors beneath a common Gkn-factor as suggested by Rolfhus and Ackerman’s Gkn model could be confirmed. While this speaks towards the cross-cultural robustness of the structure, it also implies that this structure is less sensitive to school curricula than suspected, at least in a German sample using German-specific items.

The corroborated domains can be divided into various smaller knowledge facets/categories. All domain models yielded good fits in CFAs providing structural validity evidence. Since the model of the Science domain only comprises three indicators and has zero degrees of freedom, the model fit itself is irrelevant. Nevertheless, we looked at the parameter estimates to confirm that the model makes sense at a theoretical level. Despite the good fits, cross-loadings at an item level cannot be ruled out. However, given the sample size, a test of the complete model with item indicators was not feasible. Test scale scores show varying degrees of reliability. Internal consistencies of several categories are low which most likely is due to heterogeneous content and low item variance. Hence, it is not recommended to evaluate scores at a category level. Instead, an evaluation is recommended at a total score level. Here, scores showed sufficiently high reliability estimates. 

Humanities is the largest and most heterogenous domain comprising nine knowledge categories and, based on factor loadings, is represented best by Art/Architecture. This finding is supported by the fact that six Humanities categories correlate highest with the Art/Architecture category. This result corresponds to Rolfhus and Ackerman’s (1999, p. 518) finding that Art plays a major role in defining the Humanities domain of their Gkn model. Correspondingly, Steger et al. (2019) found that, of all 34 knowledge categories, Arts and Architecture loaded highest on the Humanities factor. Interestingly, in our original design, the Medicine knowledge category was thought to belong to the Science factor instead of Humanities. However, during the construction of the DoKnow Test, the data showed that Medicine does in fact belong to the Humanities factor based on model fit and factor loadings. One explanation for this could be that nowadays many people have some basic medical knowledge. Another reason might be that the Science factor—with Physics, Chemistry, and Mathematics—does not comprise a life science category which might have been more similar to Medicine. In addition, a similar result was also found by von Stumm (2013). The second factor—named Popular Knowledge—of her knowledge model detailed above is very similar to this study’s Humanities factor (see Table 1) and contains the Medicine category as well. The Civics domain comprises four categories and is represented best by History. However, based on CFA model fits for the knowledge domain factors, Religion as one of the four categories can be assigned equally well to both the Humanities factor and the Civics factor. Nevertheless, considering the CFA model fit of the comprehensive hierarchical Gkn model comprising the Gkn-factor at the top and the three domains below, the fit indices indicate an allocation of the Religion category to Civics. This is probably also due to the fact that Religion correlates rather low with some of the Humanities categories such as Modern Literature and Sports. In conclusion, since evaluations of individual test scores are recommended at a total score level due to its high reliability, Religion was allocated to Civics. Science is the smallest and, based on factor loadings, most homogeneous domain comprising three categories. It is represented best by Physics. 

The Gkn-factor accounts for the variance of the three knowledge domains to different degrees. It explains almost the entire variance in Humanities and Civics, but only around half of the variance in Science, suggesting that the development of Science knowledge is somewhat different or requires more specific interests/abilities. This result corresponds to the finding that the Humanities and Civics domains show stronger correlations with each other than with Science. Likewise, Gustafsson and Balke (1993) found that Gc is a weak predictor of Science knowledge. 

From a theoretical perspective, the current results support the idea that acquired knowledge can be modelled in a cross-culturally consistent manner. Of course, studies in non-Western areas of the world are needed to further substantiate this hypothesis. Nevertheless, these and other previous findings can inform scale construction efforts which in turn could help to collect data able to answer the question of cross-cultural consistency. 

### 4.2. Criterion-Related Validity and Measurement Invariance

Cattell’s investment theory (Cattell 1943, 1987) was used to test the criterion-related validity of the DoKnow Test with the known-groups method (e.g., Cronbach and Meehl 1955; Hattie and Cooksey 1984). First, measurement invariance was verified at a scalar level confirming that there are no item-specific differences in difficulty between groups and allowing for mean comparisons between both samples. Overall, significant differences between the high Gf and the unselected Gf samples were found except in Humanities. As expected, the largest difference was found in Science. These results corroborate criterion-related validity for the DoKnow Test scores and are further evidence for Cattell’s investment theory. Moreover, the findings are testament for the connectedness of cognitive abilities. This connectedness in turn calls for broad assessments which are able to differentiate between specific and shared contributions. This way, the breadth of assessment directly contributes to our understanding of cognitive abilities and their interplay. 

### 4.3. Limitations and Further Research

Finally, this study shows limitations. Most importantly, the present study only provides initial psychometric evidence. It is necessary to replicate the study and establish relations with Gf by using an actual Gf measure instead of using Mensa membership as a proxy. Moreover, further convergent validity-related evidence, for example, relations that further Gc indicators such as vocabulary tests, are needed. Still, the current results can be considered sufficient to warrant further use in research settings. 

Furthermore, there was no type of proctoring during the online data collection. Following Steger et al. (2020, 2021), unproctored online testing of declarative knowledge is particularly prone to cheating as participants can easily browse through the web for correct answers since they are online already. However, considering the item difficulties observed here, this does not seem to be a strong problem. Moreover, there was nothing to gain by reaching more correct answers which might reduce the willingness to engage in cheating. Still, replicating this study should involve proctored data collection. 

Moreover, it should be examined whether the DoKnow Test measures invariantly across age groups to explore if the general structure of Gkn does not change from late adolescence through and beyond adulthood. This would be needed to fully support the use in a general population sample with the developmental questions in focus. Furthermore, it must be taken into account that this dataset has a high level of planned missing data. Future research should therefore replicate the Gkn structure with a full data set. In this context, the question of why Humanities and Civics correlate higher with each other than with Science should be investigated more closely. Moreover, during the construction, we eliminated facets due to poor model fit. Such a data driven approach can potentially limit content validity. Thus, future research is encouraged to invigorate attempts to operationalize more knowledge facets. In addition, some facet scores yielded only mediocre internal consistency estimates. While research shows that test–retest correlations are more indicative of criterion validity (e.g., McCrae et al. 2011), usage of those scores outside of research settings is currently not warranted. Lastly, due to its cultural sensitivity, the DoKnow Test is only applicable to a German population. 

## 5. Conclusions

Major achievements of this study are the confirmation of a hierarchical Gkn model structure with an operationalization of Gkn not solely based on a school curriculum and the development of a new Gkn test which is culturally sensitive and applicable to a German population including different Gf levels. Due to the prior work of Rolfhus and Ackerman (1999) and von Stumm (2013), who established their models through EFA, it was possible to confirm their theoretical model via CFA. With 16 categories and three domains, the DoKnow Test covers knowledge from different fields of academic and daily life. It provides a sound basis and future opportunity for a diverse assessment of Gkn. 

## Figures and Tables

**Table 1:** Structural Comparison of the 3 Gkn Tests.

	Rolfhus and Ackerman	DoKnow Test	von Stumm	
Humanities	**-American Literature** **-World Literature** **-Music** **-Art** **-Geography**	**-Cl. + Mod. Literature** **-Cl. + Mod. Music** **-Art/Architecture** **-Geography** -*Sports* -*Television* -*Popular* -*Medicine*	-Fashion **-Music** **-Art** -*Sports* -*Film* -*Medicine* -Health	Popular Knowledge
Civics	**-American Government** **-American History** -Law **-Western Civilization**	**-Politics** **-Economics** **-History** -Religion	**-Politics** **-Economics** **-History** **-Geography**	Academic Knowledge
Science	**-Economics** -Business/Management **-Statistics** **-Technology** -Psychology **-Biology** **-Physics** **-Chemistry**	**-Mathematics** **-Technology** **-Biology** **-Physics** **-Chemistry**	**-Science** **-Technology** **-Literature**	
Mechanical	-Astronomy -Electronics -Tools/Shop			

*Note.* Cl. = Classical, Mod. = Modern. Categories written in bold are part of all tests. Categories written in italics are part of von Stumm’s test and the DoKnow Test. Categories written normally are exclusive to the given test. The DoKnow Test categories Popular, Technology and Biology were excluded from the Gkn test in the further course due to poor psychometric quality.

**Table 2:** Knowledge Categories and Example Items of the DoKnow Test.

Category	Item	Answer
Cl. Music	*How many symphonies did Beethoven compose?*	9
Mod. Music	*Who is the lead singer of the band “Coldplay”?*	Chris Martin
Cl. Literature	*Goethe’s “the Sorrows of Young Werther” comes from which literary period or movement…?*	Sturm und Drang
Mod. Literature	*What is the name of the locomotive with which Jim Knopf and Lukas der Lokomotivführer travel?*	Emma
Art/Architecture	*To which movement in art does Salvador Dalí belong?*	Surrealism
Sports	*The five rings that make up the Olympic Games logo are blue, red, yellow, green and...*	black
Television	*Which fictitious character has been played by actors including Sean Connery, Roger Moore and Timothy Dalton?*	James Bond
Geography	*Which river is the longest river in Europe?*	Volga
Medicine	*What is the medical term for the heart’s main artery?*	Aorta
History	*The Hundred Years’ War from the 14th and 15th centuries was waged between England and…*	France
Religion	*Which book of the bible describes the journey of Moses and the Israelites out of Egypt?*	Exodus
Economics	*Who was the chairman of Deutsche Bank from 2006 to 2012?*	Josef Ackermann
Politics	*How many permanent members are on the UN Security Council?*	5
Physics	*What is “Mach 1”?*	The speed of sound
Chemistry	*Name the symbol for Lithium.*	Li
Mathematics	*When two vectors are multiplied, the result is a ...*	Scalar
Popular *	*In which city is the airport with the abbreviation CDG located?*	Paris
Biology *	*How many cells does an amoeba consist of?*	1
Technology *	*What is the most important material for the production of conventional solar cells?*	Silicon

*Note.* Cl. = Classical, Mod. = Modern. If a name was asked for, the last name was sufficient. * not included in the final test due to poor psychometric quality.

**Table 3:** Descriptive Statistics and Omegas of Knowledge Categories and the Final Test.

Knowledge Category	Number of Items	*M*	*SD*	*ω*
Humanities				
Classical Music	8	.59	.20	.46
Modern Music	8	.51	.20	.70
Classical Literature	9	.42	.18	.78
Modern Literature	6	.37	.21	.64
Art/Architecture	10	.49	.19	.86
Sports	9	.58	.17	.68
Television	7	.56	.21	.45
Geography	8	.58	.19	.51
Medicine	7	.51	.20	.54
Popular *	11	-	-	-
Civics				
History	12	.59	.17	.83
Religion	9	.54	.16	1
Economics	7	.51	.20	.33
Politics	9	.43	.17	.98
Science				
Physics	9	.67	.20	.90
Chemistry	10	.51	.20	.73
Mathematics	9	.34	.19	.74
Biology *	11	-	-	-
Technology *	11	-	-	-
Final Test	137	.50	.02	.98

*Note. N =* 1450. Scores can range from 0 to 1. * *excluded.*

**Table 4:** CFA Results for Knowledge Categories (Total Sample).

Knowledge Category	χ^2^	*df*	CFI	RMSEA	SRMR
Humanities					
Cl. Music	28.20	19	.916	.018	.037
Mod. Music	24.01	15	.968	.020	.033
Cl. Literature	10.59	8	.966	.015	.029
Mod. Literature	6.28	8	1	>.001	.026
Art/Arch.	44.49	32	.978	.016	.037
Sports	37.95 *	25	.929	.019	.045
Television	22.40 *	11	.956	.027	.037
Geography	13.65	20	1	>.001	.027
Medicine	14.48	13	.984	.009	.030
Civics					
History	90.02 **	51	.932	.023	.044
Religion	34.46	24	.950	.017	.048
Economics	13.89	14	1	>.001	.030
Politics	27.97	24	.978	.011	.033
Science					
Physics	42.27 **	26	.964	.021	.038
Chemistry	66.09 ***	32	.944	.027	.044
Mathematics	42.24 **	23	.943	.024	.042

*Note. N* = 1450. *** *p* < .001, ** *p* < .01, * *p* < .05. Cl. = Classical, Mod. = Modern, Arch. = Architecture. Robust estimation was used for CFA.

**Table 5:** CFA and Omegas of Knowledge Domains (All Samples).

Domain	χ^2^	*df*	CFI	RMSEA	SRMR	*ω*
total sample (*N* = 1450)						
Humanities	96.13 ***	19	.943	.053	.031	.72
Civics	1.14	2	1	<.001	.005	.61
Science	<.001 ***	0 ^a^	1	<.001	<.001	.72
high Gf sample (*n* = 415)						
Humanities	48.25 **	26	.919	.045	.040	.66
Civics	3.28	2	.993	.039	.017	.66
Science	<.001 ***	0	1	<.001	<.001	.64
unselected Gf sample (*n* = 1035)						
Humanities	63.82 ***	25	.961	.039	.028	.73
Civics	0.10	1	1	<.001	.002	.61
Science	<.001 ***	0	1	<.001	<.001	.66

*Note.* *** *p* < .001, ** *p* < .01. Robust estimation was used for CFA. ^a^ This is a saturated model. Therefore, model fit is perfect and cannot be interpreted. To check whether the model makes sense, we looked at loadings and correlations with other constructs.

**Table 6:** Correlation Matrix of the Knowledge Domains (All Samples).

	Humanities	Civics	Science
Humanities	1	.58/.58	.20/.31
Civics	.56	1	.25/.29
Science	.29	.29	1

*Note. N* = 1450. *n* (high Gf) = 415, *n* (unselected Gf) = 1035. Lower diagonal for total sample and upper diagonal for subsamples (high Gf/unselected Gf).

**Table 7:** CFA for General Gkn Models (All Samples).

Gkn Model	χ^2^	*df*	CFI	RMSEA	SRMR	AIC	BIC
total sample (*N* = 1450)							
One-Factor	585.88 ***	101	.867	.058	.045	37,419	37,688
Hierarchical	345.21 ***	96	.932	.042	.033	37,188	37,483
high Gf sample (*n* = 415)							
One-Factor	223.28 ***	101	.848	.054	.052	11,382	11,587
Hierarchical	165.59 ***	97	.915	.041	.043	11,331	11,553
unselected Gf sample (*n* = 1035)							
One-Factor	615.02 ***	101	.789	.070	.053	27,058	27,310
Hierarchical	306.29 ***	98	.915	.045	.036	26,755	27,022

*Note.* *** *p* < .001. Robust estimation was used for CFA.

**Table 8:** Factor Loadings for Knowledge Categories on their respective Knowledge Domain and Loadings for Knowledge Domains on the Gkn-Factor.

Category	Loadings Total Sample	Loadings High Gf	Loadings Unselected Gf
Humanities			
Classical Music	.491	.364	.503
Modern Music	.505	.497	.482
Classical Literature	.421	.240	.459
Modern Literature	.299	.233	.313
Art/Architecture	.646	.634	.658
Sports	.345	.409	.337
Television	.477	.332	.432
Geography	.519	.486	.515
Medicine	.325	.337	.288
Civics			
History	.580	.634	.575
Religion	.522	.555	.474
Economics	.464	.387	.455
Politics	.517	.616	.468
Science			
Physics	.614	.779	.808
Chemistry	.553	.301	.554
Mathematics	.593	.699	.577
*Gkn*-Factor			
Humanities	.858	.750	.909
Civics	1	1	1
Science	.545	.395	.434

*Note. N* = 1450. Loading of Civics on Gkn-factor was fixed to 1 to avoid negative residual variance. Robust estimation was used for CFA.

**Table 9:** Measurement Invariance of Gkn Test between High Gf and Unselected Gf samples.

	χ^2^	*df*	CFI	RMSEA	SRMR
Configural	442.41	192	.926	.042	.037
Metric	476.20	207	.921	.042	.043
Scalar	499.41	219	.918	.042	.044
			ΔCFI	ΔRMSEA	ΔSRMR
Configural vs. Metric			.0055	.0001	−.0058
Metric vs. Scalar			.0033	.0003	−.0013

*Note. N*_(total sample)_ = 1450, *n* (high Gf) = 415, *n* (unselected Gf) = 1035.

**Table 10:** Cohen’s *d* for Latent Means and Differences between High Gf and Unselected Gf samples.

	Humanities	Civics	Science	Gkn
*d*	0.427	0.349 ***	1.138 ***	0.510 ***
*M*	−0.08	−0.10	−0.38	−0.14
*SD*	0.18	0.27	0.34	0.27

*Note. n* (high Gf) = 415, *n* (unselected Gf) *=* 1035. *** *p* < .001. *d* = Cohen’s *d*, *M* = latent means of the unselected Gf subsample, *SD* = standard deviations. The high Gf subsample was used as reference group with their means set to 0.

## Data Availability

Data are available from corresponding author at request. This also goes for the actual DoKnow Test.

## Supplementary Materials

The following supporting information can be downloaded at: https://www.mdpi.com/article/10.3390/jintelligence11030047/s1, Tables S1/S2 CFA Results for Knowledge Categories, Table S3 Correlation Matrix of Knowledge Categories (Total Sample), Table S4 Correlation Matrix of Knowledge Categories (High Gf and Unselected Gf).
