# Supplementary material for: Measuring Domain-Specific Knowledge: From Bach to Fibonacci
Source: J Intell. 2023 Feb 28;11(3):47. doi: 10.3390/jintelligence11030047 (PMC10059680; doi:10.3390/jintelligence11030047)
Supplement: Supplementary file 1 [file jintelligence-11-00047-s001.zip › jintelligence-2194832-supplementary.pdf]

Supplementary Material

Table S1. *CFA Results for Knowledge Categories (High Gf Sample).*

Knowledge Category	χ^2	*df*	CFI	RMSEA	SRMR
		Humanities			
Cl. Music	16.14	19	1	>.001	.053
Mod. Music	19.07	18	.989	.012	.054
Cl. Literature	3.68	8	1	>.001	.030
Mod. Literature	7.14	8	1	>.001	.049
Art/Arch.	39.90	32	.950	.024	.070
Sports	25.30	24	.955	.011	.069
Television	12.14	11	.974	.016	.054
Geography	11.66	20	1	>.001	.046
Medicine	12.44	13	1	>.001	.050
		Civics			
History	49.66	50	1	>.001	.062
Religion	20.64	24	1	>.001	.073
Economics	10.43	14	1	>.001	.048
Politics	25.36	25	.996	.006	.059
		Science			
Physics	24.16	26	1	>.001	.052
Chemistry	35.32	35	.995	.005	.066
Mathematics	22.69	23	1	>.001	.056

Note. *n* = 415. Cl. = Classical, Mod. = Modern, Arch. = Architecture. Robust estimation was used for CFA.

Table S2. *CFA Results for Knowledge Categories (Unselected Gf Sample).*

Knowledge Category	χ^2	*df*	CFI	RMSEA	SRMR
			Humanities		
Cl. Music	17.02	17	1	.001	.034
Mod. Music	18.10	16	.988	.011	.034
Cl. Literature	11.05	7	.924	.024	.034
Mod. Literature	7.45	8	1	>.001	.033
Art/Arch.	32.97	32	.998	.005	.039
Sports	30.79	25	1	>.001	.049
Television	16.02	11	.975	.021	.037
Geography	16.48	20	1	>.001	.034
Medicine	15.19	13	.969	.013	.038
			Civics		
History	85.13**	52	.927	.025	.050
Religion	33.94	22	.941	.019	.054
Economics	13.21	14	1	>.001	.034
Politics	23.26	25	1	>.001	.036
			Science		
Physics	45.90**	26	.918	.027	.047
Chemistry	52.70**	31	.946	.026	.046
Mathematics	9.67	15	1	>.001	.026

Note. n = 1,035. **p<.01 Cl. = Classical, Mod. = Modern, Arch. = Architecture. Robust estimation was used for CFA.

Table S3. *Correlation Matrix of Knowledge Categories (Total Sample).*

	(1)	*(2)*	*(3)*	*(4)*	*(5)*	*(6)*	*(7)*	*(8)*
(1) Cl. Music	1							
(2) Mod. Music	.24	1						
(3) Cl. Literature	.19	.18	1					
(4) Mod. Literature	.19	.09	.16	1				
(5) Art/Architecture	.35	.33	.32	.22	1			
(6) Sports	.14	.20	.06	.06	.18	1		
(7) Television	.22	.33	.21	.14	.33	.23	1	
(8) Geography	.29	.27	.19	.12	.33	.25	.19	1
(9) Medicine	.16	.14	.12	.11	.22	.16	.16	.18
(10) History	.26	.25	.23	.18	.29	.15	.24	.31
(11) Religion	.21	.29	.23	.13	.27	.16	.24	.24
(12) Economics	.20	.21	.19	.07	.30	.20	.22	.19
(13) Politics	.18	.23	.20	.11	.28	.17	.15	.20
(14) Physics	.11	.13	.23	.08	.16	.18	.21	.13
(15) Chemistry	.13	.11	.16	.01	.14	.09	.13	.14
(16) Mathematics	.05	.11	.17	.14	.12	.11	.16	.13

	(9)	*(10)*	*(11)*	*(12)*	*(13)*	*(14)*	*(15)*	*(16)*
(1) Cl. Music								
(2) Mod. Music								
(3) Cl. Literature								
(4) Mod. Literature								
(5) Art/Architecture								
(6) Sports								
(7) Television								
(8) Geography								
(9) Medicine	1							
(10) History	.18	1						

(11) Religion	.11	.29	1					
(12) Economics	.09	.24	.20	1				
(13) Politics	.09	.33	.28	.26	1			
(14) Physics	.17	.22	.23	.16	.17	1		
(15) Chemistry	.22	.13	.14	.11	.05	.52	1	
(16) Mathematics	.11	.18	.16	.18	.21	.34	.36	1

Note. N = 1,450. Cl. = Classical, Mod. = Modern.

Table S4. *Correlation Matrix of Knowledge Categories (High Gf and Unselected Gf).*

	(1)	*(2)*	*(3)*	*(4)*	*(5)*	*(6)*	*(7)*	*(8)*
(1) Cl. Music	1	.24	.24	.18	.35	.21	.18	.29
(2) Mod. Music	.18	1	.20	.11	.31	.24	.35	.25
(3) Cl. Literature	.03	.14	1	.19	.23	.06	.10	.12
(4) Mod. Literature	.18	.04	.08	1	.23	.06	.10	.12
(5) Art/Architecture	.24	.30	.27	.17	1	.17	.34	.33
(6) Sports	.06	.27	.10	.07	.21	1	.25	.26
(7) Television	.20	.18	.13	.15	.21	.11	1	.16
(8) Geography	.20	.27	.10	.08	.31	.20	.11	1
(9) Medicine	.11	.16	.16	.14	.22	.22	.09	.11
(10) History	.28	.20	.08	.15	.27	.17	.22	.29
(11) Religion	.16	.22	.06	.05	.27	.26	.11	.26
(12) Economics	.13	.21	.10	-.02	.28	.14	.18	.16
(13) Politics	.15	.20	.02	.05	.32	.18	.07	.21
(14) Physics	.04	.02	.08	.03	.17	.17	.08	.004
(15) Chemistry	.08	-.04	.01	-.03	.03	.10	.05	.04
(16) Mathematics	-.04	.09	.09	.16	.14	.14	.11	.05

	(9)	*(10)*	*(11)*	*(12)*	*(13)*	*(14)*	*(15)*	*(16)*
(1) Cl. Music	.16	.25	.21	.21	.19	.12	.12	.04
(2) Mod. Music	.12	.26	.35	.22	.22	.16	.13	.11

(3) Cl. Literature	.10	.26	.17	.21	.21	.23	.18	.16
(4) Mod. Literature	.10	.19	.17	.09	.11	.10	-.01	.12
(5) Art/Architecture	.20	.30	.35	.30	.26	.13	.13	.09
(6) Sports	.14	.15	.11	.22	.17	.21	.09	.09
(7) Television	.14	.19	.24	.21	.14	.16	.08	.13
(8) Geography	.17	.31	.23	.20	.19	.16	.16	.14
(9) Medicine	1	.13	.16	.08	.05	.14	.20	.08
(10) History	.14	1	.29	.24	.31	.21	.12	.18
(11) Religion	.07	.38	1	.14	.19	.16	.13	.03
(12) Economics	.06	.20	.15	1	.26	.15	.10	.19
(13) Politics	.15	.39	.35	.26	1	.15	.04	.21
(14) Physics	.14	.23	.17	.13	.17	1	.46	.31
(15) Chemistry	.14	.07	.11	.08	.04	.44	1	.31
(16) Mathematics	.11	.14	.07	.12	.25	.24	.21	1

Note. *n*(high Gf) = 415, *n*(average Gf) = 1,035. Cl. = Classical, Mod. = Modern. Lower diagonal for high Gf and upper diagonal for unselected Gf.
